# An evidence-based shared decision making programme on the prevention of myocardial infarction in type 2 diabetes: protocol of a randomised-controlled trial

**DOI:** 10.1186/1471-2296-14-155

**Published:** 2013-10-19

**Authors:** Susanne Buhse, Tabitha Heller, Jürgen Kasper, Ingrid Mühlhauser, Ulrich Alfons Müller, Thomas Lehmann, Matthias Lenz

**Affiliations:** 1 Unit of Health Sciences and Education, Hamburg University, Hamburg, Germany; 2 Department of Internal Medicine III, Endocrinology and Metabolic Diseases, Jena University Hospital, Jena, Germany; 3 Institute for Neuroimmunology and Clinical MS Research, University Medical Centre Hamburg-Eppendorf, Hamburg, Germany; 4 Department of Primary Medical Care, University Medical Centre Hamburg-Eppendorf, Hamburg, Germany; 5 Centre for Clinical Studies, Jena University Hospital, Jena, Germany

**Keywords:** Diabetes mellitus, Type 2, Myocardial infarction, Primary prevention, Patient education, Patient participation, Decision making, Evidence-based medicine

## Abstract

**Background:**

Lack of patient involvement in decision making has been suggested as one reason for limited treatment success. Concepts such as shared decision making may contribute to high quality healthcare by supporting patients to make informed decisions together with their physicians.

A multi-component shared decision making programme on the prevention of heart attack in type 2 diabetes has been developed. It aims at improving the quality of decision-making by providing evidence-based patient information, enhancing patients’ knowledge, and supporting them to actively participate in decision-making. In this study the efficacy of the programme is evaluated in the setting of a diabetes clinic.

**Methods/Design:**

A single blinded randomised-controlled trial is conducted to compare the shared decision making programme with a control-intervention. The intervention consists of an evidence-based patient decision aid on the prevention of myocardial infarction and a corresponding counselling module provided by diabetes educators. Similar in duration and structure, the control-intervention targets nutrition, sports, and stress coping. A total of 154 patients between 40 and 69 years of age with type 2 diabetes and no previous diagnosis of ischaemic heart disease or stroke are enrolled and allocated either to the intervention or the control-intervention. Primary outcome measure is the patients’ knowledge on benefits and harms of heart attack prevention captured by a standardised knowledge test. Key secondary outcome measure is the achievement of treatment goals prioritised by the individual patient. Treatment goals refer to statin taking, HbA1c-, blood pressure levels and smoking status. Outcomes are assessed directly after the counselling and at 6 months follow-up. Analyses will be carried out on intention-to-treat basis. Concurrent qualitative methods are used to explore intervention fidelity and to gain insight into implementation processes.

**Discussion:**

Interventions to facilitate evidence-based shared decision making represent an innovative approach in diabetes care. The results of this study will provide information on the efficacy of such a concept in the setting of a diabetes clinic in Germany.

**Trial registration:**

ISRCTN84636255

## Background

Cardiovascular disease is the predominant life threatening complication associated with type 2 diabetes. An array of behavioural directives, such as quitting smoking, increasing exercise, normalising weight, and adhering to monitoring, dietary and medication prescriptions is imposed on patients with type 2 diabetes. Evidence on efficacy of these measures is varying and some may even do more harm than good [1]. Patients frequently feel demotivated and overloaded by the plethora of medical prescriptions. This might contribute to poor long-term adherence [2,3] even to the most effective preventative interventions such as blood pressure control [4] and use of statins [5].

International and national societies claim a “patient centred approach” such as shared decision making (SDM) in diabetes care [6,7]. SDM [8] implicates an “involvement of both the patient and the doctor, sharing of information by both parties, taking steps to build a consensus about the preferred treatment, and reaching an agreement about which treatment to implement” [9]. A prerequisite for an informed decision making process is evidence-based patient information [8,10]. In Germany however, SDM has not yet been implemented in diabetes care. Current education and counselling programmes for patients with diabetes provide rather general information, without numerical and comparative risk information [11-14].

Key objective of this project is to improve the quality of decision-making by enhancing patients’ understanding and supporting them to actively deliberate between the available treatment options. This approach is implicated by the theory of planned behaviour [15], according to which behaviour is influenced by attitudes, by subjective norms such as perceived physicians’ attitudes, and by perceived and actual individual behaviour control. Providing evidence-based information aims at strengthening behaviour control by resolving knowledge deficits. Information about probabilities of outcomes is tailored to individual risk to realign unrealistic expectations.

An informed shared decision making (ISDM) programme on the prevention of heart attack in type 2 diabetes has been developed [16,17]. In terms of a complex intervention [18], it includes a number of interdependent components that may interact with contextual factors such as the educational background of participants and the setting. Development and evaluation of these components encompassed theoretical and empirical groundwork focusing on in-depth understanding of contextual interactions and implementation processes. Details are published elsewhere [16,17,19-21].

Key component of the ISDM-programme is an evidence-based patient decision aid (DA) [17]. The DA comprises decision relevant information on the concept of benefits and harms of different preventative options, an individual risk assessment tool, and a guide to support the decision making process. A preliminary qualitative study with diabetes educators and patients [17] indicated good acceptance and suitability to support informed decision making. Implementation of the DA is considered as optimal within patient education curricula and counselling programmes. Therefore, a patient counselling module was developed [16] related to the DA (updated 08/2012) and based on current criteria of evidence-based patient information [10]. In order to simplify implementation, duration and structure have been adapted to current patient education modules [11,12]. The counselling module was piloted within the target group and iteratively optimised [16]. In order to enable diabetes educators to successfully apply the counselling, a train-the-trainer module has been developed, consisting of a video with examples and instructions, and a structured training-session.

Since piloting of the components of the ISDM programme demonstrated feasibility and acceptance, evaluation of the efficacy of the programme in a randomised-controlled trial (RCT) is required prior to implementation [18]. In addition, concurrent qualitative research is needed to monitor and to ensure reliability and validity of the intervention [22].

The reporting of this study follows current statements [23-25].

## Objectives

Primary objective is to investigate if the ISDM programme is superior to the control-intervention regarding patients’ knowledge and realistic expectations concerning benefits and harms of the available treatment options. Key secondary objective is to determine if patients in the ISDM group achieve their individual treatment goals better than patients in the control-group. Additional objectives are to assess if patients presenting high numeracy levels achieve better knowledge than patients presenting low numeracy, if social status correlates to the level of knowledge, and if patients with good knowledge achieve their treatment goals better than patients with poor knowledge.

## Methods

### Design

A parallel group, two-arm, single blinded, randomised-controlled, superiority trial with 6 months follow-up is conducted under high fidelity intervention conditions [22] comparing the ISDM programme with a control-intervention. Concurrent qualitative methods are used to explore and promote intervention fidelity [22] and to achieve in-depth understanding of implementation processes.

### Setting

Study site is the diabetes clinic at the Department for Endocrinology and Metabolic Diseases of the Jena University Hospital, Germany. In this clinic, patients are followed by diabetes educators and physicians usually twice but at least once a year. Most patients have joined a structured treatment and teaching schedule within the disease management programme (DMP). Four diabetes educators guide, motivate, and coach the patients in individual and group counselling sessions. Integral to the multidisciplinary diabetes care team, the educators develop individual plans of care, provide on-going self-management support, and analyse and adapt diet-sheets and insulin dosages. Patient characteristics, diagnoses, laboratory parameters, and prescriptions are documented in an electronic patient record system (EPRS).

### Eligibility and recruitment

Patients with type 2 diabetes between 40 and 69 years of age are eligible for enrolment if they have no previous diagnosis of ischaemic heart disease (ICD I20-I25) or stroke (ICD I63) and have participated in the DMP education programmes for patients with type 2 diabetes. Patients are eligible with HbA1c-values between 6 and 9%. This range of values is sensitive to patient participation in decision making, regarding micro-vascular prevention. Patients are excluded if they have proliferative retinopathy, chronic kidney disease stage 3 or higher [26], metastatic cancer, or are addicted to alcohol or cared by a legal guardian.

**Figure 1:**
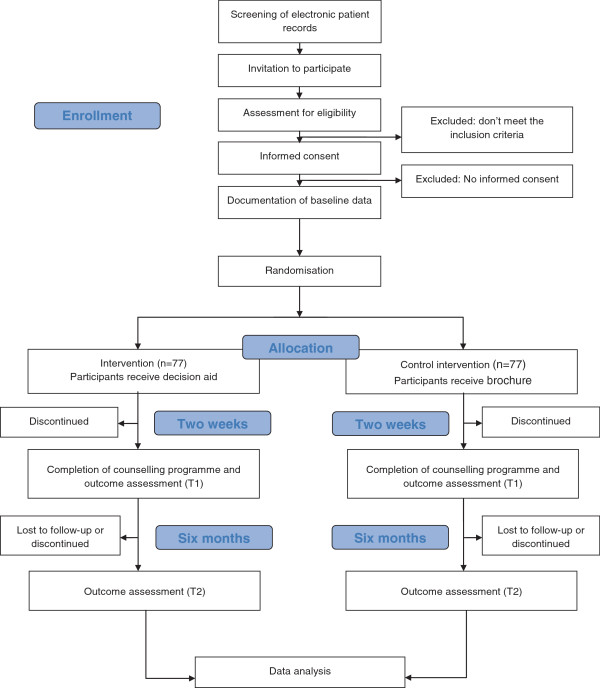
Study flow.

EPRS is screened for eligible patients (study flow Figure 1). Those are asked to participate during their regular consultation. The first patient was enrolled in March 2013. Estimated completion of recruitment is December 2013.

### Randomisation and blinding

Randomisation is performed in blocks of 8 to ensure close balance of numbers of participants in each group and sufficient numbers of participants (n = 4) in each counselling session. Randomisation sequence is generated independently by the Centre for Clinical Studies at the Jena University Hospital.

Patients are blinded to study group allocation. Allocation is concealed during data entry and analysis.

### Study interventions

#### Intervention group (ISDM programme)

The intervention (Table 1) comprises a decision aid booklet [17] and a corresponding counselling module. The counselling is provided by diabetes educators of the participating clinic. Patients are guided through the decision making process, by 1) jointly assessing the patient’s individual heart attack risk, 2) providing outcome probabilities of the available preventative treatment options, and 3) supporting to set individual goals regarding smoking cessation, glucose control, blood pressure control, and statin treatment. A physician is to be consulted if patients wish a change of treatment regimens.

**Table 1:** Characteristics of intervention and control intervention

	**ISDM programme**	**Control-intervention**
**Components**	Decision aid booklet “On the prevention of heart attack in type 2 diabetes” [17]	Brochure “Stress” [27]
	Curriculum	Curriculum
	Media: Specific wall charts, worksheets, question cards, and a magnet board	Media: Specific wall charts, worksheets, and a relaxation exercise
**Duration**	60-90 minutes	60-90 minutes
**Group size**	4 participants	4 participants

The counselling module takes about 90 minutes. Patients are asked to read and work through the DA, at least one week before the group counselling session.

For study purposes, two diabetes educators have attended the ISDM programme specific train-the-trainer module given by a research fellow of the Unit of Health Sciences and Education of the University of Hamburg. In order to ensure educational fidelity, certified diabetes educators provide the counselling sessions. To ensure fidelity of the evidence-based contents, the sessions are videotaped and analysed. If required, the research fellow gives feedback for optimisation.

#### Control group

Framing, duration and structure of the control-intervention are similar to the ISDM intervention (Table 1). In contrast to the ISDM intervention, participants are guided through a counselling session about nutrition, sports, and stress coping. The session ends after a take home relaxation exercise. At least one week before the session, all participants receive a brochure on stress management offered by health insurances [27].

### Outcomes

Primary outcome measure (Table 2) is the level of patient knowledge relating to the concept of risk, the notion of heart attack risk, and the benefits and harms of preventative treatment. Knowledge is captured by a standardised questionnaire consisting of 12 questions to assess *comprehension of risk information* and *realistic expectations*[28], plus one question to assess *numeracy*[29].

**Table 2:** Data collection

**Outcomes**	**Measures**	**No. of items**	Follow-up^*^
Knowledge	• Questionnaire developed on the basis of Bloom’s taxonomy [30] and evidence-based information on heart attack prevention in type 2 diabetes [17] (updated 08/2012)	13	T1, T2
Realistic expectations	• Questionnaire developed on the basis of ISDM-counselling	6^**^	T1, T2
Numeracy	• Numeracy test (one minute test for general population) [29]	1^***^	T1
Treatment goals	• Statin medication, levels of blood pressure and glucose control, smoking, and patient’s prioritized treatment goal are documented on a standardised form	1 for each goal	T1
Achievement of treatment goals	• Statin medication (patient medication boxes)	1	T2
	• Blood pressure (self-monitoring, standardised measure if not available)	1	T2
	• HbA1c-level (standardised measure)	1	T2
	• Smoking cessation (question by diabetes educator)	1	T2
Medication	• Variation in medication intake (statins, antihypertensive drugs, glucose-lowering agents) are documented using medical records (T1) and by verifying patients’ pill-packages (T2).	3	T1, T2

^*^ T1; at the end of the counselling session; T2; at 6 months follow-up.

^**^ 6 out of 13 questions of the knowledge test.

^***^ 1 out of 13 questions of the knowledge test.

Secondary outcome measures comprise 1) sustainability of knowledge; 2) achievement of individual treatment goals regarding the use of statins, levels of blood pressure and glucose control, and smoking; 3) achievement of treatment goals prioritised by the individual patients; 4) medication uptake from T1 to T2.

### Data collection

The EPRS of the clinic and standardised forms are used to collect baseline characteristics and outcome measures.

Baseline characteristics include age, gender, first-language, social status [31], smoking status, blood pressure, LDL-cholesterol, total cholesterol, HbA1c, current medication, and previous participation in diabetes patient education programmes.

Baseline risks of myocardial infarction are calculated by using a risk assessment tool based on age, smoking status, and clinical parameters. The tool is derived from the Framingham function and calibrated [32] to the average heart attack risk in the German population.

Knowledge is assessed directly after the counselling session and at 6 months follow-up. The individual treatment goals regarding smoking cessation, glucose control, blood pressure control, and statin treatment are assessed after the counselling session. Achievement of treatment goals is assessed during a consultation at 6 months follow-up. Current medication is monitored by verifying patients’ pill-packages. Smoking status is assessed by using a standardised interview question. Current blood pressure values (mean of 2 weeks) are extracted from patient diaries. If values are not available, blood pressure is measured before counselling by a nurse applying a standardised procedure (after 5 minutes seated position). Glucose control is assessed by measuring HbA1c-levels [33].

### Data synthesis

All statistical analyses are carried out according to the intention-to-treat principle. Missing data will be imputed using the method of multiple imputation if feasible. All analyses will be computed using IBM SPSS Statistics 19.0 for Windows.

Baseline characteristics are described using means of standard deviation (± SD) or frequencies, as appropriate according to the level of measurement.

#### Primary outcome

Unpaired t-tests will be used to compare mean scores of knowledge and realistic expectations directly after counselling.

#### Secondary outcomes

Unpaired t-tests will be used to compare mean knowledge scores at follow-up. Fisher’s exact test will be used to compare proportions of sufficient knowledge after counselling and at follow-up. Participants will be rated as having sufficient knowledge if they correctly answered at least 8 out of 12 questions. Unpaired t-tests will be used to compare average individual differences between planned and achieved values of blood pressure and HbA1c. Fisher’s exact tests will be used to compare the groups regarding rates of individual goal achievement (yes/no): statin choice, smoking status, blood-pressure (defined as reaching 80% to 120% of the goal), and HbA1c (defined as reaching 80% to 120% of the goal). Mann Whitney U-Test will be used to compare the medication uptake from T1 to T2 (increase/unchanged/decrease).

#### Sub-group analyses

Two groups of participants will be defined regarding their level of knowledge (sufficient / insufficient). Fisher’s exact tests will be used to assess if knowledge is associated with the level of numeracy (yes/no) and with achievement of goals (yes/no). T-test for paired samples will be used to assess differences in knowledge between T1 and T2 in the intervention group. Analyses of variance (ANOVA) will be used to assess if knowledge is associated with age (groups 40–49; 50–59; 60–69). Spearman’s Rho correlation coefficient will be used to assess if knowledge is associated with social status.

Mann Whitney U-tests will be calculated to assess if heart attack risk and social status are associated with the achievement of goals (yes/no).

### Sample size

We hypothesise patients in the ISDM group to reach higher levels of knowledge and realistic expectations. Based on pilot studies conducted during the development phase of the knowledge test, we estimate that participants having received the intervention will reach 70% correct answers in the knowledge test, whereas those having received the control-intervention will reach 50% correct answers. We calculate the sample size providing 80% power to detect an absolute difference of 20% between the intervention and the control group, using a two-tailed t-test at the 5% level of significance. Estimating a standard deviation (SD) of 0.4 in both groups, data on about 64 patients per group (128 participants) need to be included in data-analysis. Estimating a non-responder/drop-out rate of about 15%, 154 participants will need to be recruited for randomisation.

### Intervention fidelity and process evaluation

The efficacy of the ISDM programme may depend on the complex interaction between components (e.g. provider training and patient counselling strategies) and terms and conditions of the setting. Strategies to monitor and promote intervention fidelity [22] focus on study-design, provider training, delivery and receipt of intervention, and enactment of treatment skills (Table 3).

**Table 3:** Intervention fidelity strategies

**Category **[22]	**Goals **[22]	**Elements of the ISDM study**
Design of study	• Ensure same treatment dose within conditions.	• Curriculum and media are standardised for both study arms
	• Ensure equivalent dose across conditions.	• Intervention and control-intervention are similar in framing, duration and structure
	• Plan for implementation setbacks	• For both intervention and control-intervention, two diabetes educators are trained to ensure the completion of the counselling sessions
Training providers	• Standardize training	• All diabetes educators are trained in standardised train-the-trainer sessions
	• Ensure provider skill acquisition	
	• Minimize “drift” in provider skills	• Educational material is standardised
		• Optimal patient counselling is demonstrated
	• Accommodate provider differences (adequate level of training, skills, experience and professional background)	• Providers practise counselling under supervision of a research fellow and subsequent feedback
		• Providers assess the patient knowledge questionnaire to ensure skill acquisition
Delivery of intervention	• Control for provider differences	• Counselling sessions are video-taped, constantly analysed, and fed back by a research fellow
	• Reduce differences within treatment	• Counselling protocol: deviation from curriculum (duration, material use, content, didactics) is documented
	• Ensure adherence to protocol	
	• Minimize contamination between conditions	
Receipt of intervention	• Ensure participant knowledge	• Questionnaire cards at the end of the counselling session. If there are difficulties in understanding, the diabetes educator discusses and corrects the answer and repeats the information
	• Ensure participant ability to use cognitive skills	
	• Ensure participant ability to perform behavioural skills	
Enactment of treatment skills	• Ensure participant use of cognitive skills	• Patients set individual treatment goals for heart attack prevention
	• Ensure participant use of behavioural skills	• If patients make treatment decisions that differ from their current treatment goals a physician is consulted for clarification

Underlying processes are monitored to explore why the intervention has worked or not. Video-taped counselling sessions are constantly analysed and fed back to maintain and optimise the fidelity of education and contents.

### Ethical approval

The study protocol was approved by the ethics committee of the Jena University Hospital. Written participant information about study objectives and procedures are given to eligible patients. Standardised forms are used to document informed consent.

### Confidentiality

In order to maintain data privacy, pseudonyms are used to combine data sets (baseline and follow-up data) and to identify data if patients withdraw informed consent. The pseudonym list is kept under lock at the Jena University Hospital. Participant information is kept in locked file cabinets. Computer files are code-locked to prohibit unauthorized access.

## Discussion

The planned study aims to determine the efficacy of a complex counselling programme [16] regarding patient knowledge, realistic expectations and achievement of individual treatment goals. The used approach is innovative: Certified diabetes educators provide evidence-based patient information on heart attack risk and prevention, discuss the individual notion of that information, and initiate a shared decision making process. Patients are motivated to set individual goals choosing the treatment options most important to them.

The study is conducted in the context of a diabetes out-patient clinic, which allows high standardization to monitor and maintain intervention fidelity. Moreover, qualitative methods are used to explore processes and barriers of implementation. A corresponding cluster-RCT is planned to assess the effectiveness of the ISDM-programme in the setting of primary care-practices.

## Abbreviations

DA: Decision aid; DMP: Disease management programme; EPRS: Electronic patient record system; ICD: International classification of diseases; ISDM: Informed shared decision making; RCT: Randomised controlled trial; SDM: Shared decision making.

## Competing interests

The authors (SB, TH, JK, IM, UAM, TL and ML) declare that they have no competing interests.

## Authors’ contributions

This study protocol was carried out in collaboration between all authors. SB, JK, ML and IM are involved in the study design. TH and UAM are involved in the planning, coordination and management of data acquisition at study site (diabetes out-patient clinic). TL has contributed to the statistical planning of the study. SB and ML wrote the first draft of the manuscript. IM, JK, TH, and UAM substantially contributed to the draft of the manuscript. IM and JK critically revised the manuscript. ML, JK, UAM and IM conceived the study and applied for funding. All authors have read and approved the final version of the manuscript.

## Authors’ information

SB is research fellow at the MIN Faculty, Unit of Health Sciences and Education, University of Hamburg, Germany. ML and JK are senior researchers at the MIN Faculty, Unit of Health Sciences and Education, University of Hamburg, Germany. IM is Professor of Health Sciences and Education, University Hamburg, Germany, and specialist in Internal Medicine, Diabetology and Endocrinology.

TH is a research fellow at the Department for Internal Medicine, Endocrinology and Metabolic Diseases in Jena, Germany. TL is biostatistician at the Centre for Clinical Studies of the Jena University Hospital. UAM is Professor for internal medicine and head of the Department for Endocrinology and Metabolic Diseases of the Jena University Hospital.

## Pre-publication history

The pre-publication history for this paper can be accessed here:

http://www.biomedcentral.com/1471-2296/14/155/prepub
